# Myeloma research on the move

**DOI:** 10.1038/s41408-021-00550-z

**Published:** 2021-09-17

**Authors:** Heinz Ludwig

**Affiliations:** Wilhelminen Cancer Research Institute, First Department of Medicine, Clinic Ottakring, Vienna, Austria

**Keywords:** Myeloma, Translational research

In the last years, remarkable progress has been achieved in our understanding of the biology and management of multiple myeloma (MM). Some of these advancements and possible future directions are addressed below and summarized in Table 1.

**Table 1:** Advances in multiple myeloma research with clinical implications.

	New findings	Possible consequences
Genomic data	Whole-genome sequencing revealed two types of MGUS: 1. with myeloma-defining events already present at diagnosis, with high risk for progression 2. A stable myeloma precursor condition with low risk of progression	Genomic definition of multiple myeloma
		Possible scenarios:
		1. Stable myeloma precursor condition
		2. Multiple myeloma-CRAB^neg^
		3. Multiple Myeloma-CRAB^pos^
Treatment of HR SMM/early MM	Genome sequencing defined two types of multiple myeloma as indicated above	Biology-based definition will select patients who benefit most from early treatment initiation (not necessarily HR/SMM patients defined by present algorithms)
Mass spectrometry	Higher sensitivity for detection of monoclonal proteins (MP)	Mass spectrometry (MS) will become the preferred technology for the detection and quantification of MPs. The higher sensitivity will reveal a higher prevalence of MGUS MS may be used in addition to NGS or NGF, or a sole method for MRD assessment
Diffusion weighted whole-body MRI	Higher sensitivity for detection of focal lesions, diffuse infiltration, and extramedullary disease compared to PET/CT	Likely to become the preferred imaging technology, but published standards for acquisition and reporting of WB MRI must be adhered to
ASCT	Improves PFS in all patients, in high-risk patients, OS is prolonged as well	Will remain standard, particularly in cytogenetic high-risk patients until the introduction of more effective therapies
Daratumumab (and likely other anti CD38 antibodies)	Two studies in NTE patients show already an OS benefit when combined with chemotherapy backbones for first-line therapy, in TE patients daratumumab combinations resulted in deeper responses, higher MRD^neg^ rates, and longer PFS. For OS longer FU is required. In later treatment lines, daratumumab combinations resulted in significant benefit, as well as daratumumab single-agent therapy	Anti-CD38 antibody combinations have become standard for first-line therapy
MRD status	The predictive value of MRDneg has been documented in an extensive meta-analysis. NGS (FDA approved) and NGF have a sensitivity of 10^-6^, MRD^neg^ is associated with a survival advantage	MRD assessment is already standard in clinical trials, and likely will be approved as a proxy for OS Sustained MRD^neg^ ultimate goal of therapy
Maintenance therapy	Addition of anti-CD-38 antibodies or proteasome inhibitors to lenalidomide maintenance improves outcome	Drug combinations will be used for maintenance therapy but long term follow up data are required for further recommendations
Antibody conjugates, BiTEs, and other antibodies	Belantamab mafodotin showed significant single-agent activity in RRMM, several BiTEs showed high response rates in heavily pretreated patients	This drug class will be eagerly taken up in clinical practice, because of the substantial activity and easy access as drugs may be available on- the-shelf. Caveats are the toxicity and limited PFS. Will be combined with various drugs and moved to earlier lines of therapy
CAR-T cells	The CARTITUDE trial showed 97% ORR and 77% PFS rate after one year, similar slightly less impressive results were reported in KarMMa trial	Aside from anti-CD 38 antibodies the second most important game-changer. Will be evaluated for first-line therapy. Modifications of the CAR-T cells will further increase efficacy. Modified allogeneic CAR-T cells will become on-the-shelf products

## Genomic data suggest rethinking the definition of myeloma

Whole-genome sequencing revealed two types of MGUS: A progressive myeloma precursor condition defined as a clonal entity in which myeloma-defining genomic events have already been acquired at the time of diagnosis and which is associated with a high risk of progression to MM; and an MGUS with a stable myeloma precursor condition, in which myeloma-defining genomic events are rare and which follows an indolent clinical course with a low risk of progression [1]. In those patients, branching evolution may still lead to progressive disease, but this seems to be rare and usually takes longer.

These findings may inaugurate a new era of a genomic definition of monoclonal gammopathies, abandoning the arbitrarily defined categories MGUS, SMM, and MM, which depend on the disease burden but not on the underlying biology. This may be replaced by a genetic definition that may distinguish between three types: A. stable precursor condition of multiple myeloma, B. multiple myeloma—CRAB^neg^, and C. multiple myeloma—CRAB^pos^.

## Treatment of high-risk smoldering multiple myeloma or of early myeloma?

As indicated above, the presently applied parameters of high-risk SMM do not reflect the genomic structure of individual patients and thus preclude optimal patient selection for early treatment. Further studies will path the way for a biology-based definition or a combination of these factors with conventional parameters of those patients that have already acquired the genetic machinery for malignant progression and thus would benefit most from early treatment initiation. As many experts consider myeloma still as being incurable, the question of optimal patient selection and timing for treatment initiation is highly pertinent. In the future, this question will become less relevant, because of the better differentiation between risk groups and further improvements in treatment outcome.

## Mass spectrometry will replace conventional methods for M-protein assessment

Mass spectrometry (MS) shows higher sensitivity for the detection of monoclonal proteins (MP) compared to the conventional electrophoretic techniques [2] and will become the preferred technology for the detection and quantification of monoclonal proteins with profound implications. More individuals with a tiny MP will be detected, thus increasing the global prevalence of MGUS. Sequential studies will confirm that MGUS can be transient like other lymphoproliferative diseases such as alpha heavy chain disease [3], and this phenomenon may be more frequent in individuals with small MPs. The Spanish PETHEMA group [4] found a significant concordance between patients defined as MRD^neg^ either by NGF or MS, and a similar prognostic accuracy of both techniques for PFS. In addition, they identified a small group of either MS^neg^−MRD^pos^, and MS^pos^−MRD^neg^ patients. Whether MS will be used in addition to MRD testing, or whether MS may even replace conventional MRD testing needs to be explored. In case of similar sensitivity and predictability, MS could even become the preferred MRD test because it enables frequent serial testing, which may inform on both reaching and sustaining MS^neg^, as well as losing MS^neg^ status as an early indication of relapse.

## Whole-body MRI may become the preferred imaging technology

Present imaging guidelines in MM recommend low dose CT, PET/CT, and whole-body MRI (WB MRI) irrespective of their individual advantages and limitations. This may change if results of a recent prospective randomized study [5] showing higher sensitivity of diffusion-weighted WB MRI for detection of myeloma bone and extramedullary disease compared to PET/CT will be confirmed, and if diffusion-weighted WB MRI findings inform equally well or even better about imaging assessed myeloma response and its correlation with progression-free survival as PET/CT. An important requirement for achieving optimal MRI results is the standardization of acquisition and reporting of diffusion-weighted WB MRI. Further analysis showed a fivefold higher sensitivity of diffusion-weighted WB MRI (81.7% vs 16.7%) for detection of diffuse bone marrow infiltrations compared to PET-CT. All patients with high-risk cytogenetics had diffuse marrow infiltrations and most of them were missed by PET/CT. This study might become practice-changing recommending diffusion-weighted WB MRI for detection of myeloma bone and extramedullary lesions. This would also impact on the selection of the imaging technique for exclusion of residual disease in NGS or NGF-defined MRD^neg^ patients.

## Autologous stem cell transplantation, particularly in high-risk disease, will remain standard for the near future

Autologous stem cell transplantation (ASCT) has greatly improved treatment outcome before the introduction of new agents and continues to be a valuable choice in the era of novel agents. ASCT during induction therapy improved PFS over conventional chemotherapy in practically all trials, but OS was found to be similar in most studies [6]. Discussion is ongoing whether ASCT can be replaced by novel treatments in standard-risk patients. Several trials have established its benefit in patients with high-risk (HR) cytogenetics with the improvement of both PFS and OS [7]. The most recent confirmation of the efficacy of ASCT comes from the Italian FORTE study [8]. This trial showed superiority of KRd over KCd for induction therapy and most importantly, a significant improvement of the depth of response and PFS with eight cycles of KRd plus ASCT compared to 12 cycles of KRd. The advantage of ASCT was seen in patients with standard-risk cytogenetics, in those with one, and with two or more cytogenetic high-risk abnormalities. Hence, ASCT will remain standard of care, particularly in cytogenetic HR patients until further improvements in first-line therapy.

## Daratumumab—chemotherapy combinations have become the new standard for first-line therapies

Daratumumab is a game-changer for MM therapy, active as single-agent therapy in heavily pretreated patients, synergistic with several chemotherapy backbones in later lines of therapy, and finally a valuable combination partner for first-line treatment regimens with an already documented survival advantage in patients not eligible for transplantation (ALCYONE and MAIA) [9]. Studies in transplant-eligible patients (CASSIOPEIA) [10] showed an impressive prolongation of PFS, but for OS, longer follow-up will be required. In elderly patients and in studies with relapsed refractory myeloma (POLLUX and CASTOR) [9], daratumumab was given until progression or intolerance, while in the transplant studies, daratumumab maintenance is usually restricted to 2 years. Long follow-up is needed for documentation of a possible survival benefit.

## MRD status will become a surrogate parameter for overall survival

New technologies, in particular next-generation sequencing (NGS) and next-generation flow (NGF), have successfully been adapted to enable the detection of one myeloma cell within one million nucleated bone marrow cells. Patients achieving MRD^neg^ at a threshold level of 10^−6^ show prolonged survival compared to MRD^pos^ patients [11]. Achieving MRD^neg^ at least once is already associated with a survival advantage, but sustained MRD^neg^ is the ultimate goal. Remarkably, patients with HR cytogenetics achieving MRD^neg^ seem to fair equally well as MRD^neg^ standard-risk patients. A comprehensive meta-analysis has confirmed the close association between MRD^neg^ and survival in practically all treatment scenarios, including younger and older patients, first or later lines of therapy, and different risk groups [11]. This relationship was even noted in the BELLINI study with discordant PFS and OS results [12]. Overall survival is still considered the gold standard for evaluation of the impact of any new therapy, but with an increasing survival expectancy of MM patients, meeting overall survival as primary study endpoint becomes increasingly difficult to achieve, highlighting the need for a surrogate marker, such as sustained MRD^neg^.

## Standard lenalidomide maintenance therapy is challenged by new drugs and combinations

Lenalidomide maintenance after ASCT is still the gold standard, but trials with proteasome inhibitors and anti-CD38 antibodies, either as single-agent or in combination with lenalidomide, are ongoing. Carfilzomib added to lenalidomide resulted in improved PFS in all cytogenetic risk groups, with the exception of patients with ampl1q in the FORTE study [8], compared to single-agent lenalidomide, thus showing that carfilzomib may be a valuable combination partner for lenalidomide, but the requirement for intravenous infusion limits the treatment duration of carfilzomib. Recent results from the CASSIOPEIA trial [13] indicate significant activity of daratumumab maintenance treatment in patients without daratumumab during induction therapy, a finding which would speak against continuous exposure, but PFS2 tended to be superior in patients exposed both during induction and maintenance therapy to daratumumab. In the GRIFFIN study [9], patients randomized to daratumumab-lenalidomide maintenance showed significantly higher sCR and MRD^neg^ rates compared to the lenalidomide arm. Presently, new drugs such as CELMoDs and BiTEs are evaluated as maintenance treatments.

## BiTEs, other antibodies, and antibody toxin conjugates

Belantamab Mafodotin shows remarkable single-agent activity in RRMM patients [14], which has prompted its evaluation in combination with several backbones. Keratopathy as a side effect is an issue, but progress has been made in mitigating this complication by dose reduction, longer treatment intervals, or both. Bi-specific T cell engager (BiTEs) have two binding sites: one targeting an immune cell and the other a myeloma membrane antigen [15]. Clinical studies showed high anti-myeloma activity with response rates up to 83% in heavily pretreated patients. Treatment with BiTEs can be associated with a previously uncommon side effect profile including CIRS and neurotoxicity. The ease of administration of BiTEs and their ready availability on-the-shelf gives them an advantage over cellular therapies.

## CAR-T cells and other cellular therapies

With the development of CAR-T cell therapy, an old dream of hematologists became true. Harnessing the patient’s own immune system can induce marked tumor responses in far advanced, heavily pretreated patients. A response rate of 97% with high rates of MRD^neg^ disease and a PFS rate of 77% after one year has been reported in the CARTITUDE-1 study [16], but the persistence of CAR-T cells and long-term myeloma control is still an issue. Several developments making CAR-T cells even more efficient and better tolerable are ongoing. Bispecific CAR-T cells may reduce the risk for antigen escape, fully human CAR constructs will improve persistence, allogeneic CAR-T cells may, after adequate gene editing to remove the T cell receptor and disruption of the MHC complex, be available on-the-shelf. Importantly, trials are already ongoing comparing upfront CAR-T cell therapy with ASCT.
